# Cronkhite-Canada Syndrome: Sustained Clinical Response with Anti-TNF Therapy

**DOI:** 10.1155/2018/9409732

**Published:** 2018-07-02

**Authors:** S. A. Taylor, J. Kelly, D. E. Loomes

**Affiliations:** ^1^Division of Gastroenterology, University of Toronto, Toronto, ON, Canada; ^2^Department of Pathology, University of British Columbia, Victoria, BC, Canada; ^3^Division of Gastroenterology, University of British Columbia, Vancouver, BC, Canada

## Abstract

Cronkhite-Canada syndrome (CCS) is a rare, nonfamilial syndrome that occurs in the sixth to seventh decades of life. It is characterized by acquired gastrointestinal polyposis with an associated ectodermal triad, including alopecia, onchodystrophy, and hyperpigmentation. CCS is characteristically a progressive disease, with a high mortality rate despite medical interventions. Disease complications are typically secondary to severe malnutrition, malignancy, GI bleeding, and infection. CCS is believed secondary to immune dysregulation; however, the underlying etiology remains to be determined. Treatment for CCS is largely anecdotal, and randomized controlled therapeutic trials are lacking due to the rarity of the disease. Aggressive nutritional support in conjunction with immunosuppression has been used previously with inconsistent results. In this report, we describe the presentation and diagnosis of a case of CCS and report encouraging treatment response with anti-TNF therapy.

## 1. Introduction

Cronkhite-Canada Syndrome (CCS) is a rare, nonfamilial hamartomatous polyposis syndrome that is characterized by polyps distributed throughout the stomach and colon (90%), small bowel (80%), and rectum (67%) with characteristic esophageal sparing [1, 2]. This condition was first described by Cronkhite and Canada in 1955, and the incidence is now estimated to be one per million persons per year [3]. It is a disease of middle age with an average age of diagnosis in the early 60s, and it is more common in males (3 : 2) [4]. Interestingly, the majority of cases in the literature have been reported in Japan.

The typical clinical presentation is varied, illustrated by Goto, in a epidemiologic retrospective study of 110 cases of CCS reported in Japan [3]. The most common presenting symptoms include hypogeusia (40.9%), diarrhea (35.4%), abdominal discomfort (9.1%), alopecia (8.2%), and xerostomia (6.4%) [3, 5]. Intestinal bleeding and intussusception are rare but potentially lethal complications of CCS [6]. The classic CCS dermatological triad includes alopecia, skin hyperpigmentation, and onychodystrophy.

The differential diagnosis for CCS includes a number of other polyposis syndromes including Cowden's disease, Peutz-Jeghers syndrome, Turcot syndrome, and juvenile polyposis syndrome; however, compared to juvenile polyposis syndrome, CCS polyps are less pedunculated and demonstrate inflammatory cell infiltration in the lamina propria with associated edema [7]. Conventional adenomatous polyps have also been reported in CCS. Despite high coincident rates of gastrointestinal and colorectal carcinoma, it remains unclear if CCS is a premalignant condition or if this is associated with conventional adenoma-carcinoma sequence progression.

Diagnosis of CCS is clinical, based on clinical presentation, endoscopic findings, and histopathology. There is no consensus for an underlying etiology of pathogenesis; however, immune dysregulation has been implicated as this condition is commonly identified in patients with lupus, hypothyroidism, and rheumatoid arthritis [2, 8, 9]. Additionally, serology commonly shows antinuclear antibody positivity [10]. More recently, gastric and colonic CCS polyps have been shown to immunostain IgG4 positive, raising the possibility that IgG4 may be involved in CCS pathogenesis [11].

Medical treatment for CCS is not based on firm science as controlled randomized therapeutic trials have not been possible due to the rarity of the disease. One of the most important mainstays of treatment is aggressive nutritional support with a high protein diet, hyperalimentation, and fluid and electrolyte replacement [12]. Antiacid measures including histamine receptor antagonists, proton pump inhibitors, and cromolyn have been used, particularly in patients with biopsies demonstrating eosinophilia [13].

Systemic immunosuppression is the most common medical treatment tried, yielding anecdotal and inconsistent results [14]. A number of studies have reported that timely corticosteroid therapy can facilitate endoscopic regression of the polyposis syndrome resulting in nodular mucosa with a cobblestone appearance, but it is unclear if this translates to a change in the natural history of the disease. There is no consensus for appropriate dose and duration of glucocorticoid therapy [4, 14, 15]. Immunomodulators including azathioprine, calcineurin inhibitors, and cyclosporine have been tried with mixed success [8, 16, 17].

Recently, Watanabe et al. have described a patient with steroid-refractory CCS exhibiting a dramatic clinical and endoscopic improvement with infliximab (Remicade) therapy [6]. Here, we report the fourth case report in the English literature describing a prototypical case of CCS which was successfully treated with an anti-TNF.

## 2. Case Report

### 2.1. Clinical Presentation

A 76-year-old male was referred to the emergency department in May 2016 for significant unintentional weight loss of approximately 57 kg and associated chronic nonbloody watery diarrheal illness in the preceding 18 months. Medical history was notable for prostate cancer curatively treated in 2012, gout, a remote transient ischemic attack, osteoarthritis, and bilateral cataracts. In the months prior to presentation to Gastroenterology, an extensive medical workup performed as an outpatient was negative for prostate cancer recurrence, new malignancy, autoimmunity, or an identifiable malabsorption syndrome including celiac disease and pancreatic insufficiency.

**Figure 1:**
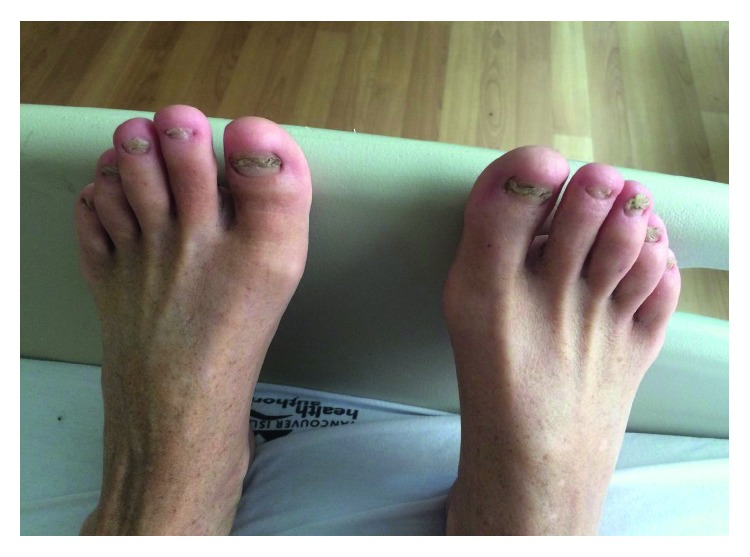
Onchodystrophy of toenails.

**Figure 2:**
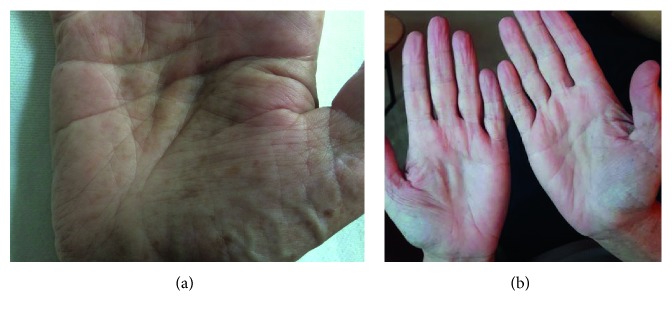
(a) Hyperpigmentation of hands before therapy. (b) Resolution of hyperpigmentation 9 months following therapy with infliximab.

The patient also noticed onycholysis in both his hands and feet (Figure 1), followed by hyperpigmentation of his hands (Figure 2), soles of his feet and legs, and abdomen. In addition to the nonbloody diarrhea, the patient reported a severe change in taste, early satiety, chronic heartburn, and nonspecific abdominal pain. He denied a history of fever, cough, night sweats, or abdominal pain. There was no family history of gastrointestinal malignancy or similar disorder.

Physical examination demonstrated profound cachexia with a weight of 50.9 kg and a BMI 16.5. Generalized sarcopenia was noted. The abdomen was scaphoid and nontender with no hepatosplenomegaly. Nonscarring alopecia was seen on the scalp, dystrophic nail changes were identified in both the hands (Figure 1) and feet, skin hyperpigmentation was noted primarily involving the palms (Figure 2), dorsal aspects of fingers, face, and limbs, as well as sexual pattern hair loss of the abdomen, groin, and axillary hair. No cervical, inguinal, or axillary lymphadenopathy was identified. The rest of the physical exam was unremarkable.

### 2.2. Investigations

Complete blood count was notable for a mild normocytic anemia (hemoglobin 119 g/L (reference range, 130–175 g/L) and mild eosinophilia of 0.82 g/L (reference range, 0–0.35 g/L)). Serum albumin was low at 28 g/L (reference range, 35.0–55.0 g/L). Serum electrolytes platelet count, white count, renal, liver enzyme and function tests, lipase and total protein, serum immunoglobulins, CRP, and TSH were normal. PSA was undetectable. Autoantibodies, including antinuclear antibody, antineutrophil cytoplasmic antibody, and rheumatoid factor (RF) were undetectable as were serologic tests for HIV, hepatitis, syphilis, and Lyme disease. Serum protein electrophoresis exhibited a modest elevation in kappa free light chains (23.0 g/L) but a normal kappa/lambda ratio was not consistent with a monoclonal gammopathy. There were no extended nutrient deficiencies with lead, copper, zinc, B12, or iron. Fecal elastase, stool culture, *C. difficile*, ova and parasites, and fecal leukocytes were negative. Stool for occult blood was positive.

**Figure 3:**
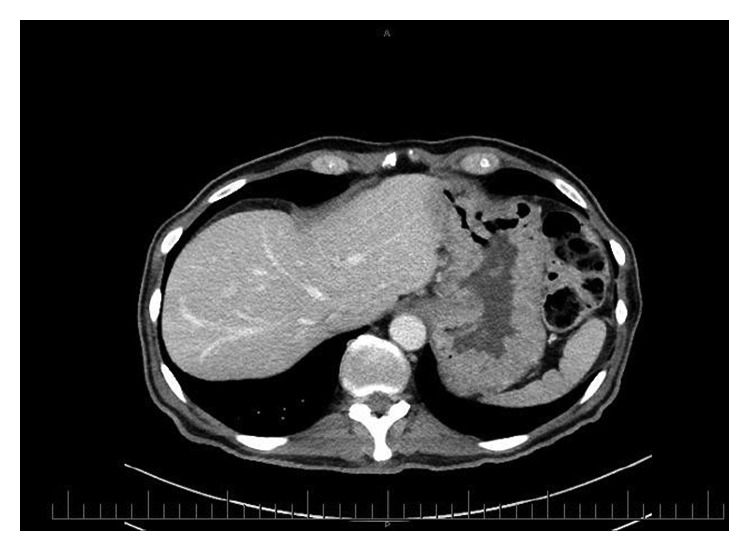
Abdominal CT on admission demonstrating hypertrophic gastric folds.

Abdominal computed tomography (CT) demonstrated extensive gastric and duodenal mucosal fold thickening (Figure 3).

**Figure 4:**
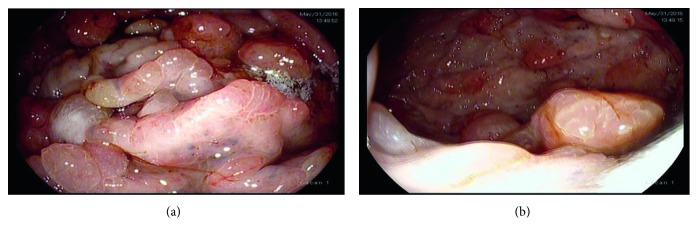
Endoscopic findings of the upper gastrointestinal tract at the initial diagnosis. (a) The stomach revealing mucosal edema and hypertrophic gastric villi and (b) multiple semipedunculated gastric polyps.

Upper endoscopy demonstrated florid gastric and duodenal polyposis, with thickening of gastric folds and “carpet-like” semipedunculated gastric and duodenal polyps ranging from 5 mm to 20 mm (Figures 4(a) and 4(b)). Histologically, the duodenal polyps showed edematous mucosa with variably dilated and branching glands, foci of gastric foveolar metaplasia, and blunted or absent intestinal villi. The inflammatory cell content of the lamina propria was mildly increased with prominent eosinophils.

**Figure 5:**
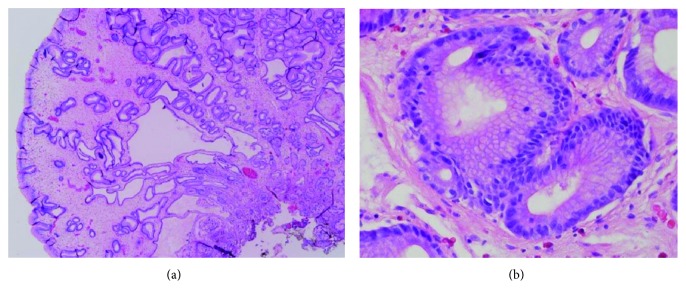
(a, b) Irregular glands with focal cystic dilation, severe edema of the lamina propria, and a sprinkling of eosinophil leukocytes.

Where native intestinal-type surface epithelium remained, it showed a mild increase of intraepithelial lymphocytes and an occasional intraepithelial eosinophil. There was no subepithelial collagen deposition. The gastric polyps were also a characteristic of Cronkhite-Canada syndrome. The foveolar glands were elongated, irregular, and focally dilated. The lamina propria was widely expanded by edema with an infiltrate of eosinophils and mononuclear cells (Figures 5(a) and 5(b)). Helicobacter organisms were not identified in gastric or duodenal specimens. The involvement of the duodenum and gastric antrum in this process ruled out Menetrier's disease which is typically confined to the gastric body.

Based on these clinical, endoscopic, and histopathologic features, a diagnosis of Cronkhite-Canada Syndrome was made.

### 2.3. Outcome and Follow-Up

Due to near complete inability to take in enteral intake from severe early satiety and subjective global assessment of severe malnutrition, TPN was initiated in conjunction with a short course of methylprednisolone, followed by a tapering prednisone regimen starting at 50 mg per day. Azathioprine was also initiated at 75 mg daily. A jejunostomy tube was placed under radiological guidance to provide enteral nutrition, and a high protein formula was used for caloric requirements, as the patient was unable to take in more than a few tablespoons at a time.

Approximately six weeks after discharge, during the course of continued outpatient evaluation, the patient exhibited a worsening of his diarrheal illness accompanied by fever and progressive abdominal pain. Stool testing was positive for C difficile, and oral vancomycin was initiated with satisfactory clinical response. After several clinical relapses on vancomycin taper, the patient was advised by infectious diseases to continue suppressive vancomycin 125 mg PO daily.

Several months into steroid taper, the patient developed polyuria, polydipsia, and hyperglycemia which had not been present at higher steroid doses. Insulin was initiated with reversion to normoglycemia.

Despite adequate enteral caloric intake and immunosuppression, the patient continued to experience progressive weight loss, failure to thrive, and ongoing diarrhea (*C. difficile* toxin-negative). Based on a successful recent case report, off-label infliximab was employed [6]. Typical induction and maintenance infusions of infliximab were initiated with a regimen of 5 mg/kg at weeks 0, 2, and 6 followed by maintenance regimen of 5 mg/kg every 8 weeks thereafter. Remicade level was within the accepted range at week 14. Azathioprine was initiated with infliximab, at the beginning of therapy to prevent antibody formation to the anti-TNF.

The patient did not have an immediate initial response, and due to nausea, azathioprine was discontinued after 3 months. Azathioprine metabolites showed a 6-thioguanine of 106 pmol/8 × 10^8^ RBC in the nontherapeutic range (230–400) and an undetectable 6-methyl mercaptopurine, appropriate for combination therapy with infliximab. Therefore, azathioprine was discontinued.

At 4 months from induction, the patient began to have nail regrowth, improvements in taste, and a modest improvement in diarrhea and weight.

Eight months following induction therapy with infliximab, bowel hygiene was significantly improved with approximately two formed movements daily. The patient was able to resume eating and drinking, and weight had increased by 10 kg. The patient also noted an improved sense of taste. Physical examination showed hair regrowth on the scalp, abdomen, and axillary and pubic regions in addition with improved proximal nail bed health. Hyperpigmentation was globally improved (Figure 2(b)). Laboratory values were within normal range.

Repeat upper endoscopy 9 months after initiation of anti-TNF showed notable improvement in gastric distention; however, there was persistent polyposis and no obvious pathological improvement in inflammatory cell infiltrate.

## 3. Discussion

CCS is a rare clinical entity characterized by diffuse gastrointestinal polyposis and unique ectodermal changes of alopecia, hyperpigmentation, and nail dystrophy. It is a near-uniformly progressive disease. Additionally, the 5-year mortality has been estimated as high as 55%. On review of the English literature, there are 59 cases of CCS treated with prednisone with a clear response defined by clinical improvement in symptoms of malabsorption or endoscopic improvement defined primarily by polyp regression.

Including our own case, 17% of CCS cases have been identified as corticosteroid resistant. Azathioprine has shown utility in maintaining remission of disease in 5 patients, with a median remission period of 4.5 years [8]. Other reported successful treatments for CCS include calcineurin inhibitors, cyclosporine, and TNF antagonists. Anti-TNF therapy has been reported in three cases with clinical response dictated by symptom improvement and weight gain, as well as polyp regression in 2 of these patients [6].

Here, we report a fourth CCS case partially responsive to anti-TNF therapy. This is also the first case of CCS reported in Canada [6, 17, 18]. Anti-TNF therapy was associated with clinical improvement in weight, appetite, taste, alopecia, and ectodermal changes. Regression of polyposis has not occurred, unlike a previously reported case [6].

There has been no evidence of gastrointestinal or colorectal cancer thus far. Frequent endoscopic surveillance will be continued given high rates of concomitant colorectal and gastric cancer reported in this patient population.

Further prospective studies are needed to assess the effectiveness of steroid-sparing treatment given the superior side effect profile of these agents compared with high-dose glucocorticoid therapy.

## 4. Conclusion

In summary, we present a prototypical case of CCS with marked clinical response and partial endoscopic response after treatment with aggressive enteral nutrition and azathioprine and infliximab combination therapy.
